# Third Molars on the Internet: A Guide for Assessing Information Quality and Readability

**DOI:** 10.2196/ijmr.4712

**Published:** 2015-10-06

**Authors:** Kamal Hanna, David Brennan, Paul Sambrook, Jason Armfield

**Affiliations:** ^1^ PhD Candidate, Australian Research Centre for Population Oral Health (ARCPOH) School of Dentistry The University of Adelaide Adelaide, South Australia Australia; ^2^ Professor, Australian Research Centre for Population Oral Health (ARCPOH) School of Dentistry The University of Adelaide Adelaide, South Australia Australia; ^3^ Consultant OMFS, Department of Oral and Maxillofacial Surgery Adelaide Dental Hospital The University of Adelaide Adelaide, South Australia Australia; ^4^ Associate Professor, Australian Research Centre for Population Oral Health (ARCPOH) School of Dentistry The University of Adelaide Adelaide, South Australia Australia

**Keywords:** DISCERN, health information online, Health on the Net Code, readability, Scientific Information Quality Scale, website affiliation, website content analysis, wisdom teeth

## Abstract

**Background:**

Directing patients suffering from third molars (TMs) problems to high-quality online information is not only medically important, but also could enable better engagement in shared decision making.

**Objectives:**

This study aimed to develop a scale that measures the scientific information quality (SIQ) for online information concerning wisdom tooth problems and to conduct a quality evaluation for online TMs resources. In addition, the study evaluated whether a specific piece of readability software (Readability Studio Professional 2012) might be reliable in measuring information comprehension, and explored predictors for the SIQ Scale.

**Methods:**

A cross-sectional sample of websites was retrieved using certain keywords and phrases such as “impacted wisdom tooth problems” using 3 popular search engines. The retrieved websites (n=150) were filtered. The retained 50 websites were evaluated to assess their characteristics, usability, accessibility, trust, readability, SIQ, and their credibility using DISCERN and Health on the Net Code (HoNCode).

**Results:**

Websites’ mean scale scores varied significantly across website affiliation groups such as governmental, commercial, and treatment provider bodies. The SIQ Scale had a good internal consistency (alpha=.85) and was significantly correlated with DISCERN (*r*=.82, *P*<.01) and HoNCode (*r*=.38, *P*<.01). Less than 25% of websites had SIQ scores above 75%. The mean readability grade (10.3, SD 1.9) was above the recommended level, and was significantly correlated with the Scientific Information Comprehension Scale (*r*=.45. *P*<.01), which provides evidence for convergent validity. Website affiliation and DISCERN were significantly associated with SIQ (*P*<.01) and explained 76% of the SIQ variance.

**Conclusion:**

The developed SIQ Scale was found to demonstrate reliability and initial validity. Website affiliation, DISCERN, and HoNCode were significant predictors for the quality of scientific information. The Readability Studio software estimates were associated with scientific information comprehensiveness measures.

## Introduction

Wisdom teeth removal is the most commonly performed oral surgical procedure [1]. In addition to patients needing to make a decision regarding whether or not to remove asymptomatic wisdom teeth [2,3], other decisions need to be made regarding anesthetics options, treatment pathways and associated costs, operation timing, and expected recovery [1]. Patients who undergo third molars (TMs) extraction prefer to receive detailed procedural information [4]. Providing those patients with detailed high-quality information is not only medically and legally important in making an informed decision, but also might improve their participation in the process of shared clinical decision making. This might, in turn, improve patient satisfaction and treatment outcomes [5].

It is not always possible to provide adequate information for patients suffering from TMs problems, because it might be limited by the available consultation time allocated to each patient, given the fact that clinics are often overbooked [6]. The busy nature of oral surgery clinics may hinder surgeons from adequately explaining the provided information, a finding suggested by Ferrús-Torres et al [7]. Lack of sufficient information from professional sources and limitations of information leaflets [8] can result in patients seeking online sources to satisfy their information demands and often before consultation [9]. While the Internet plays an increasing role in dental patient education [10], the quality of online health information varies significantly across websites [11,12]. Therefore, it is argued that clinicians should guide their patients to credible online health resources.

There can be a potential limitation in the current clinical practice in referring patients to high-quality Internet resources due to clinicians’ lack of time and/or lack of knowledge [13,14]. In addition, the lack of dentists’ ability to discuss the retrieved conflicting Internet-related information with their patients may affect the patient-dentist relationship [10]. To provide patients with guidance in navigating the Internet, clinicians could use the findings from website content analysis studies. However, only a small number of dentally related studies exist and none have covered wisdom tooth problems. The lack of content analysis studies means the absence of an evidence base with which clinicians might be able to guide their patients to credible Internet-based resources. Furthermore, identifying predictors for scientific information quality (SIQ) could make the process of identifying high-quality online resources easier and less time consuming. However, clinicians also need to ensure that the high-quality Internet resources they identify are understandable by their patients.

Understanding health information is a major domain in health literacy, allowing patients to make appropriate health-related decisions [15]. Patients with higher levels of health literacy have been found to have a better oral health status [16]. To ensure that consumer health information is understandable by the average patient, some health authorities require this information to be at Grade 8 reading level or less (13-14 years of age) [17]. Readability grades are calculated using different readability formulas [18] and are mainly based on word/sentence length and number of syllables per word. These provide a reading grade in relation to the US schooling system, which is set as a reference for readability grading. However, it would be useful to know which of these formulas has the highest association with information comprehensibility. A number of software applications and websites provide a readability-grade estimate for digital documents. Among these software applications, Readability Studio Professional 2012 [19] has been used in some studies [20,21] to calculate readability grades using different formulas. However, readability-grade estimates produced by Readability Studio software need to be assessed for their validity to measure information comprehensibility.

The aims of this study were to (1) develop and validate a scale that measures SIQ; (2) evaluate the quality and readability of online health information concerning TMs problems; (3) validate the Readability Studio Professional 2012 software for measuring comprehensibility of online information; and (4) explore factors that could predict the SIQ of online health information.

## Methods

### Website Sampling and Filtering

**Figure 1:**
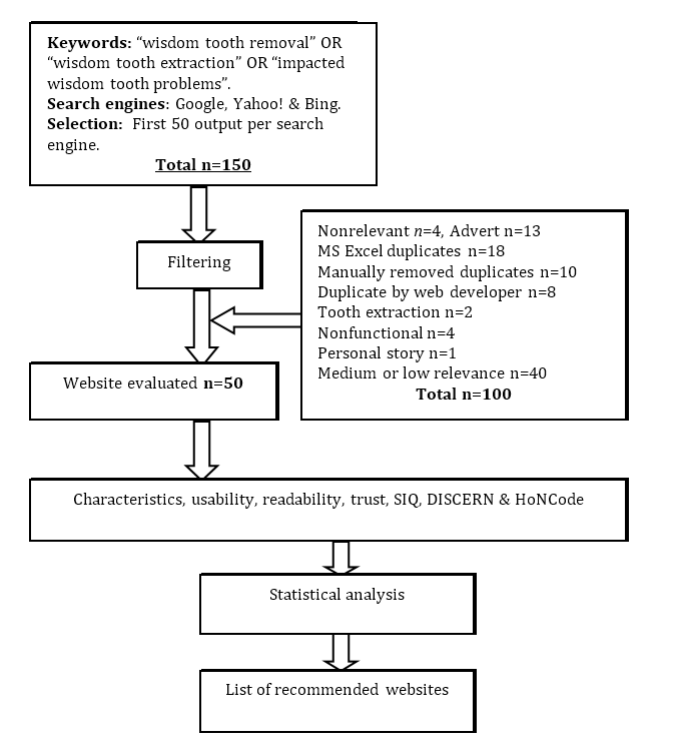
Website content analysis flowchart.

To identify high-quality online resources, a cross-sectional sample of websites was selected on October 14, 2013, using advanced search options in Google, Yahoo!, and Bing search engines, with output limited to English language, any location, and specific phrases in the page title. The 3 phrases used were “wisdom tooth removal” OR “wisdom tooth extraction” OR “impacted wisdom tooth problems.” The first 50 results of each search engine output were selected after excluding websites identified as advertisements. A total of 150 websites were initially included. Websites were then filtered by removing duplicates and were reviewed for their relevance as a source for patient information. During this stage, nonfunctional, nonrelevant news articles or blogs were excluded. If a website was found to be relevant, it was categorized as having high, medium, or low relevance based on reporting the predetermined information sections of the SIQ Scale. Only websites of high relevance, according to this classification, were selected for content analysis. Figure 1 shows the flowchart for website sampling and filtering. This review was conducted by KH for consistency and eliminating the need for providing training.

### Characteristics of Website

A number of website characteristics that might have an association with the quality of provided information were determined. Website affiliation (to which organization the website belongs to) was recorded as governmental, educational institute, treatment provider (hospital/medical or dental practice), nonprofit organization, commercial [22], or other group. There was an open section that was then coded into blogs, hub pages, wiki (like Wikipedia), or news. As content editing could play a role in information quality, websites were categorized into either “open access” or “open content” where the type of content editing was reported for coding. Information delivery format was recorded, as previous research shows the importance of multimedia use to engage patients of low literacy [23]. Information formats were recorded as a multiple response set that included “text within the webpage,” “word/PDF,” “images,” “cartoon animations,” “audio,” “real example,” and “other,” with an open section to enable adding comments, which later were coded into themes. Information communication method was recorded to identify the prevalence of each method. Information communication methods were recorded as a multiple response set that included “fact sheet,” “question and answer (Q&A),” “story,” and “other” with an open section that was coded into other types of information formats.

### Website Evaluation

To assess different quality aspects of websites under evaluation, several scales were used [24]. Quality aspects included scales assessing usability, accessibility, trustworthiness, readability grade [18], scientific information comprehensiveness, scientific information reporting, scientific information referencing, SIQ, and online health information credibility (Health on the Net [25] and DISCERN [26]). The sum of these scales formed the website total score, which was considered as a collective measure of website quality.

#### Usability Scale

The Usability Scale consisted of items that were partially based on the Minervation Tool (LIDA [27]). These items include registration/subscription to review the information, website navigability, and search ability and were given a score that ranged from 1 to 3 for each item based on the response. For Web 2.0 applications support such as Facebook, Twitter, LinkedIn, and G+, responses were collected as a multiple response set to provide a score that was then trichotomized based on percentile distribution. Usability Scale scores range from 4 to 12.

#### Accessibility Scale

A single-item Binary Scale that was used as a proxy for compliance with Web Content Accessibility Guidelines [28], with scores being 1 for “No” and 3 for “Yes” to increase item weight in the website total score.

#### Trust Scale

The Trust Scale was developed for this study and comprised a 4-item Binary Response Scale measuring trust in a website. Items for this scale were display of the Health on the Net (HoN) seal [25], as it is the most frequently used online consumer health information quality seal, validity of the HoN seal using the HoN toolbar, display of other quality seals, and display of planned review date as an indication for maintaining information currency. Items for this scale were scored 1 for “No” and 3 for “Yes” to increase the scale weight within the website total score. The scale scores range from 4 to 12.

#### Mean Readability Grades

Mean readability grades were computed using Readability Studio Professional 2012 that provides readability grades estimates based on 6 different formulas recommended for the health care industry, which are FORCAST, Fry, Gunning Fog, New Fog, Raygor Estimate, and SMOG. Text from websites was extracted to MS Word (Microsoft, Redmond, WA, USA) where they were prepared for evaluation by the software. In addition, videos were transcribed by the author (KH).

#### Scientific Information Comprehension Scale

The Scientific Information Comprehension (SI Comprehension) Scale was developed specifically for this study. It comprises a 9-item scale that measures the understandability of each section of the scientific information shown in Table 1. Items for this scale were scored on a 5-point Likert-like scale ranging from 1 “difficult to understand” to 5 “easy to understand.” If the item did not exist on the website, it was reported as missing.

**Table 1:** Evaluation criteria for scientific information quality concerning wisdom tooth problems.

Criteria (assessed on a 5-point Likert-like scale: range from 1 for poor to 5 for excellent)	Description
Overview	Number of third molars (TMs), age of eruption, and etiology of impaction
Presentation	Mild pericoronitis to severe infection of facial spaces, swelling, trismus, periodontitis, decay, cyst, or tuners with incidence. No evidence supports the association between TMs and late teen crowding.
Diagnosis and investigations	Diagnosed by a dentist/oral surgeon, medical and dental history, clinical and radiographic examination, and other radiographs in high-risk TMs
Treatment options	Retain functional TMs, symptomatic TMs with untreatable conditions or associated with pathology should be removed, no evidence supports the removal of asymptomatic impacted TMs, shared decision making. Anesthetic options (local anesthetic, intravenous sedation, or general anesthetic). Pathway (minor oral surgery, hospital day case, or hospital inpatient).
Risk and benefits	Incidence of risks associated with retaining TMs, general surgical risks (pain, bleeding, swelling, etc), anatomical-related risks (numbness of lip or tongue, oroantral fistula), rare risks (tuberosity/mandible fracture)
Surgical procedure	Draping, anesthesia, flap, bone removal, tooth sectioning, tooth removal, socket irrigation, socket inspection, bone filing, suture, and gauze pack
Postoperative care and recovery	Postoperative instructions, how to control pain, bleeding, swelling, infection, and dry/infected socket. Information about diet and oral hygiene. Expected recovery.
Costs associated with the treatment	Depend on pathway: direct cost (surgeon, anesthetize, and/or hospital fees), indirect cost (time off work), insurance information
More information for intravenous sedation and general anesthesia/dental anxiety management	Conscious sedation (oral, inhalation and intravenous sedation), general anesthetic

#### Scientific Information Reporting Scale

The Scientific Information Reporting (SI Reporting) Scale is a 9-item binary scale that was developed for this study based on reporting information topics, which can be found on the assessed website. Items for this scale were scored 1 if the information section was covered and 0 if the information section was not covered in the examined website. Full scale scores range from 0 to 9. The SI Reporting Scale was used to identify websites of high relevance as a source of information.

#### Scientific Information Referencing Scale

The Scientific Information Referencing (SI Referencing) Scale is a 9-item Binary Scale that was developed for this study to measure referencing different information sections on the assessed website. Items for this scale were scored 1 if the information section was referenced and 0 if the information section was not referenced, and the full scale scores again range from 0 to 9.

#### Scientific Information Quality Scale

The SIQ Scale is a 9-item Likert-like scale, which was developed to assess various aspects of information that should be provided to patients, based on literature review and authors’ experience in the field (Table 1). Each item was scored on a scale ranging from 1 “poor” to 5 “excellent” against the predetermined criteria for online information concerning TMs problems that was created by this study’s authors using the best available evidence (see Multimedia Appendix 1). Domains for this scale included overview (introduction), presentation, diagnosis, treatment options, risks/benefits, procedural information, postoperative care and recovery, costs, and more information about anxiety control. If the item did not exist on the website, it was reported as missing. The SIQ Scale scores range from 9 to 45.

#### Online Consumer Health Information Credibility Tools

##### Health on the Net Code Scale

This was a 14-item scale that was developed by authors [24] based on the criteria for providing the HoN seal [25]. Each item had 3 response options, namely, the website was “not complying” with Health on the Net Code (HoNCode) (scored 1), the website was “partially complying” with HoNCode (scored 2), and the website was “fully complying” with HoNCode (scored 3). The HoNCode consists of the following 8 principles: authorship, complementary information, maintaining privacy, appropriate referencing of information sources, claim policy, transparency, disclose funding source, and clear advertising policy. The HoNCode Scale scores range from 14 to 42.

##### DISCERN Scale

This is a 16-item scale developed by Charnock [26] to assess the credibility of printed consumer health information and was validated for assessment of online consumer health information [29]. Each item was scored 1 for a “definitive no,” 2-4 for “partial yes” (based on reviewer’s judgment), or 5 for a “definitive yes.” The DISCERN items are grouped into 3 main groups: Questions 1-8 are related to reliability of information, Questions 9-15 are related to specific treatment choices, and Question 16 provides an overall quality assessment of the information. The DISCERN Scale scores range from 16 to 80.

#### Website Total Score

The website total score was used as a measure of the total website quality. It was calculated as an unweighted sum of website usability, trust, SIQ, scientific information comprehensiveness, scientific information referencing, scientific information reporting, accessibility, DISCERN, HoNCode Scales, and the reverse-coded mean readability grade. The website total scores range from 57 to 222.

#### Reviewer’s Comments

To allow the evaluator (KH) to provide qualitative feedback on the assessed websites, the researcher commented on areas of biased/unbalanced information. In addition, the researcher commented on factors that might affect information readability and the recommended treatment options. These comments were then coded into themes and subthemes for analysis.

### Data Analysis

Data were analyzed using IBM SPSS Statistics for Windows version 22.0 (IBM, NY, USA) [30]. Frequencies of websites characteristics were calculated. Means, SDs, and quartile distributions were also calculated for each scale. The internal consistency using Cronbach alpha of each scale was calculated. Pearson *r* correlation coefficients were calculated between SIQ Scale, DISERN, and HoNCode. In addition, Pearson *r* correlation between the mean readability grade and the reverse-coded SI Comprehension Scale was measured in an attempt to establish convergent validity. The associations between website affiliation and websites scale scores were tested using one-way analysis of variance (one-way ANOVA) with Tukey post hoc tests.

To explore predictors for SIQ scores, linear regression was performed after creating dummy variables for website affiliation groups. A block of website affiliation dummies (Model 1) was entered in linear regression, where the “other” group was used as a reference category. In Model 2, DISCERN was added, and in Model 3, DISCERN was removed and replaced by the HoNCode score while statistically controlling for website affiliation. Websites were ranked according to their SIQ score and to their total (unweighted) score. The correlation between the 2 ranking orders was examined using Spearman ranking correlation.

The website reviewer’s (KH) comments were analyzed using NVivo 10 [31] where comments were coded into themes and subthemes. These themes included biased/unbalanced information (subthemes included areas of biased/unbalanced information), factors affecting information readability (subthemes included repetition, terminologies use, image labeling), and the recommended treatment options (subthemes included obtaining a second opinion, prophylactic removal of all TMs, removal of only symptomatic ones, removal of symptomatic, and seriously think about asymptomatic ones). Cross-tabulation of codes’ frequency by the website affiliation group was obtained for unbalanced/biased information, the recommended treatment and factors affecting information readability, and then weighted according to the percentage of representation of the website affiliation group within the sample.

## Results

### Websites Characteristics and Their Usability

Of the 50 websites available for content analysis, a majority of the reviewed websites (54%, 27/50) were related to a treatment provider after adding 1 website to this group from the “educational institute” group that has a teaching hospital attached to it. A total of 7 of the 50 (14%) websites were related to commercial websites, and governmental and nonprofit organizations websites were equally represented (8%, 4/50). There were 7 “other” group websites (hub pages, blogs, news, and wiki, 14%). A combination of text and image was the most commonly used information format (40%, 20/50). Question and answer was the most predominant information communication method either alone (34%, 17/50) or in combination with fact sheets (22%, 11/50).

Most websites were open access (74%, 37/50), and the most common form of content editing was posting comments (14%, 7/50). All websites were accessible without either registration or subscription. A majority of websites were judged easy to navigate (62%, 31/50) while slightly above half of the websites (52%, 26/50) had no search facility. Facebook (23% of Web 2.0 applications, 28/121) and Twitter (20% of Web 2.0 applications, 24/121) were the most commonly used Web 2.0 applications.

### Scientific Information Quality

The developed SIQ Scale had good internal consistency (Cronbach alpha=.85). Furthermore, the SIQ scores were significantly correlated with DISCERN scores (*r=*.81, *P*<.01) and HoNCode (*r=*.38, *P*<.01). Less than 25% of the evaluated websites had SIQ scores above 75% of the maximum scale score. The overview section was the most reported information section, whereas the cost information section was the least reported.

### Information Credibility Tools

DISCERN had high internal consistency (Cronbach alpha=.91), whereas that for HoNCode was slightly lower (Cronbach alpha=.80). DISCERN and HoNCode were significantly correlated with each other (*r*=.71, *P*<.01) and both scales were significantly correlated with the SIQ Scale (as mentioned earlier).

### Association of Website Affiliation With Website Scores

One-Way ANOVA showed a significant association between website affiliation and SIQ (*F*_4,45_=4.8, *P*<.01), DISCERN scores (*F*_4,45_=4.8, *P*<.01), and HoNCode score (*F*_4,45_=8.8, *P*<.01). SIQ had an observed power of 90% or over for each of them and had moderate effect size estimates. Website affiliation was also significantly associated with the other scales (Usability, Trust, SI Referencing, and SI Comprehension) except for the mean readability grade where no significant difference was found. Tukey post hoc tests showed that the SIQ mean scores of the “other” website affiliation group was significantly lower than commercial websites (*P<*.01) and governmental website (*P=*.01). Table 2 shows the significant association of websites scales mean scores and total score with website affiliation groups.

**Table 2:** Quality and readability scores by website affiliation.

	Website affiliation^a^										
	Commercial		Treatment provider		Government		Nonprofit organization		Other		
	Mean	SD	Mean	SD	Mean	SD	Mean	SD	Mean	SD	*P*-value significance
Scientific Information Quality (SIQ)	32.6_a_	9.5	26.0_a_	7.8	34.8_a_	10.9	29.0_a_	9.8	17.0_b_	7.3	.01
DISCERN	62.3_a_	9.3	46.0_b_	10.7	59.3_a_	17.6	61.8_a_	12.1	44.1_b_	13.5	<.01
Health on the Net Code	35.4_a_	4.3	26.8_b_	3.7	35.3_a_	2.5	35.8_a_	4.9	30.7_a_	7.8	<.01
Mean readability grade	10.3_a_	0.9	10.6_a_	1.4	10.4_a_	3.7	11.3_a_	2.9	9.0_a_	2.7	.34
SI Comprehension	9.9_a,c_	1.3	12.8_b_	2.6	9.8_a,b,c_	2.2	13.5_a,b_	7.6	8.3_c_	4.5	.01
SI Referencing	10.6_a_	2.4	9.1_b_	0.5	9.8_a,b_	0.5	11.8_a_	3.1	10.1_a,b_	2.3	.01
Trust	5.0_a_	1.4	4.1_b_	0.4	4.8_a,b_	1.0	4.5_a,b_	1.0	4.0_b_	0.0	.03
Usability	10.3_a_	1.4	8.4_b_	1.1	10.5_a_	0.6	9.8_a_	1.3	10.4_a_	1.4	<.01
SI Reporting	16.9_a_	1.6	16.2_a_	1.4	17.0_a_	2.0	16.3_a_	2.2	13.9_b_	2.1	.01
Accessibility	1.0_a_	0.0	1.1_a_	3.0	1.5_b_	0.6	1.0_a_	0.0	1.0_a_	0.0	.02
Total score	153.8_a_	26.1	114.3_b_	22.3	152.6_a_	29.9	144.9_a_	26.9	114.0_b_	25.4	.02

^a^Values in the same row and subtable not sharing the same subscript are significantly different at *P*<.05 in the two-sided test of equality for column means. Cells with no subscript are not included in the test. Tests assume equal variances.

### Predictors for Scientific Information Quality

Linear regression models (Table 3) showed that website affiliation alone (Model 1) significantly explained 21% of the adjusted *R* ^2^ of SIQ scores. Governmental websites had the highest (*B*) coefficients (*B*=17.75, *P<*.01) in comparison to the “other” group that was set as a reference category. After controlling for website affiliation (Model 2), DISCERN scores were found to be significantly associated with the highest SIQ (*B*=.60, *P*<.01). Because DISCERN and HoNCode are measuring a close construct, DISCERN was removed from the regression equation and replaced by HoNCode in Model 3. While controlling for website affiliation, HoNCode was found to significantly predict the SIQ (*B*=.63, *P*=.02). A regression residual scatter plot showed a random distribution while the P-P plot of the observed and the predicted values of the SIQ scores showed a good model fit (data not presented).

**Table 3:** Scientific Information Quality score prediction models.^a^

Model		Unstandardized coefficients		95% CI for B		Standardized coefficients	*t*	Significance (*P* value)	Model summary		
		*B*	Standard error	Lower bound	Upper bound	Beta			Adjusted *R* ^2^	*R* ^2^ change	Significance *F* change
1	Constant	17.00	3.16	10.64	23.36		5.38	<.01	.21	.28^b^	.01
	Commercial	15.57	4.47	6.58	24.57	.58	3.49	<.01			
	Treatment provider	8.96	3.53	1.85	16.08	.48	2.54	.02			
	Governmental	17.75	5.24	7.20	28.30	.52	3.39	<.01			
	Nonprofit organization	12.00	5.24	1.45	22.55	.35	2.29	.03			
2	Constant	-9.50	3.16	-15.87	-3.14		-3.01	<.01	.76	.51^c^	<.01
	Commercial	4.68	2.71	-0.77	10.13	.17	1.73	.09			
	Treatment provider	7.83	1.96	3.87	11.79	.42	3.99	<.01			
	Governmental	8.68	3.05	2.55	14.82	.25	2.85	.01			
	Nonprofit organization	1.43	3.09	-4.80	7.66	.04	0.46	.65			
	DISCERN	0.60	0.06	0.48	0.72	.85	10.09	<.01			
3	Constant	-2.45	8.49	-19.56	14.67		-0.29	.76	.29	.09^d^	.02
	Commercial	12.59	4.41	3.70	21.47	.47	2.85	.01			
	Treatment provider	11.43	3.50	4.38	18.48	.61	3.27	<.01			
	Governmental	14.88	5.11	4.59	25.17	.43	2.91	.01			
	Nonprofit organization	8.81	5.14	-1.54	19.16	.26	1.72	.09			
	Health on the Net Code Scale	0.63	0.26	0.11	1.16	.39	2.45	.02			

^a^The “other” website affiliation group was used as a reference category.

^b^ *R* ^2^change for Model 1: It is change from a null model.

^c^ *R* ^2^change for Model 2: It is a change from Model 1.

^d^ *R* ^2^change for Model 3: It is a change from Model 1.

### Information Readability Grades and Comprehension

**Figure 2:**
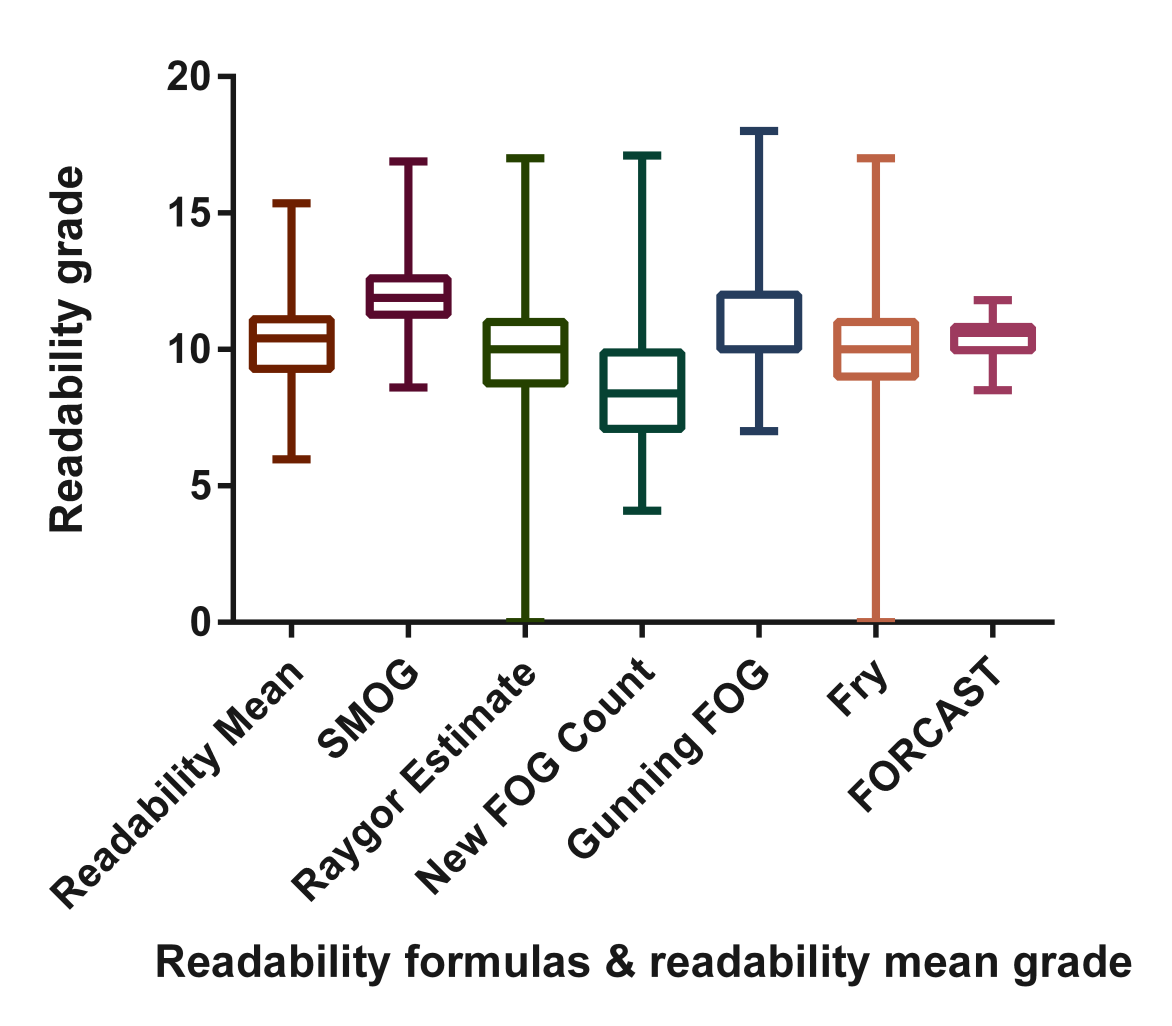
Box plot of readability grades and mean readability grade.

The mean (SD) readability grade (Figure 2) was 10.3 (1.9). Nonprofit organization websites had the highest mean readability grade, whereas the “other” websites had the lowest mean readability grade (Table 2). There was no significant difference in the mean readability grade among website affiliation groups. One-way ANOVA of readability grade estimates (FORCAST, Fry, Gunning Fog, New Fog Count, Raygor Estimate, and SMOG) grouped by website affiliation showed no significant difference except for FORCAST, which was found to be significantly different (*F*_4,45_=3.2, *P=*.02). Figure 2 shows box plots of 6 different readability grades and the mean readability grade calculated using Readability Studio. After reverse coding of the Scientific Information Comprehension Scale scores, the New FOG readability grade has the highest significant association with it among the used readability formulas (*r*=.48, *p*<.01). In addition, the reverse-coded SI Comprehension Scale scores were found to be significantly correlated with the mean readability grade produced by Readability Studio Professional 2012 (*r*=.45, *P*<.01).

### Websites’ Ranking

Websites were ranked according to their SIQ scores. Results showed that the Bupa-UK website [32] had the highest SIQ, followed by that of the NHS-UK [33]. Ranking websites according to their total unweighted scoring showed that Bupa-UK had the highest total score followed by Animated-Teeth [34]. Spearman ranking correlation between both ranking orders were significantly correlated (*r*=.81, *P*<.01).

### Analysis of Reviewer’s Comments

The comment section was analyzed using thematic analysis. Biased or unbalanced information provided was coded. TMs and late teen crowding were the most frequently reported biased information (41% of reported biased/unbalanced information, 13/31). Forcing patients to undergo “sleep dentistry” (16% of reported biased/unbalanced information, 5/31) was an example of unbalanced information, where information providers limited the anesthetic options to general anesthesia or sedation without providing local anesthetic as an option. The treatment provider group was associated with the highest frequency of biased/unbalanced information (74% of reported biased/unbalanced information, 23/31), which was also confirmed by the weighted frequencies of biased/unbalanced information across different website affiliation groups.

Comprehensibility of information was affected by the use of terminologies without explanation (28% of reported readability issues, 10/35), and/or the use of illustrations that were incorrectly labeled (5% of reported readability issues, 2/35), or inadequately labeled (5% of reported readability issues, 2/35), or sometimes not relevant at all (11% of reported readability issues, 4/35). In addition, poor information presentation and organization (11% of reported readability issues, 5/35) played an important role in the ability of finding information. Furthermore, repetition was found in some of the reviewed websites (11% of reported readability issues, 5/35).

The most frequently reported treatment option was the removal of symptomatic wisdom teeth and to seriously consider removal of asymptomatic ones (30% of reported treatment options, 7/23), while 4 websites (17% of reported treatment options) recommended the prophylactic removal of all wisdom teeth to “get peace of mind.” A number of websites (28% of reported treatment options, 6/23) recommended the removal of only symptomatic ones. There were instances where patients were advised to get a second opinion (17% of reported treatment options, 4/23) before making a treatment choice related to their wisdom teeth. Coronectomy (removing the crown and retaining the root) as a treatment option for high-risk wisdom teeth was rarely mentioned.

## Discussion

### Preliminary Findings

In this study, we aimed to provide a guide to assess the quality and readability of online health information with an application on Internet-related information concerning TMs problems using a scale developed for this purpose. The study also identified a shortlist of high-quality resources that might be recommended by clinicians to patients having TMs problems. Because online resources are dynamic, the researchers explored predictors for SIQ that might be used for a quick and easy identification of high-quality online resources.

To identify high-quality resources, a search was carried out using 3 common search engines (Google, Yahoo!, and Bing), and 3 keywords thought to be used by an average patient. While some authors have claimed that patients do not normally go beyond the first 25 results [35], the number of websites included per search query ranged from 10 to 100 websites. Accordingly, we decided to include the first 50 websites per search engine query. The number of websites remaining for thorough evaluation in this study was considered reasonable according to existing literature where the websites included for final analysis ranged from 21 [36] to 67 [37] with a mean of 38 websites per study. In addition, the observed power for the association between website affiliation and website scores was found to be high.

Internet information was delivered using mainly question and answer format either alone or together with fact sheets. Preferences of dental patients in relation to information delivery format need further investigation as there is a knowledge gap in the existing literature in this area. In addition, treatment providers should consider using online forums on their websites supported by health professionals to allow for a better engagement with patients [38]. Despite the importance of multimedia use in patient education [23], a combination of text and images was the most commonly used method of presenting information. There were instances where images were not related to the discussed topic, or were inadequately or incorrectly labeled. It is argued that the use of multimedia is associated with high costs due to professionalism, especially if these websites are for small businesses. Efforts should be made by professional and public health organizations to make multimedia available with permission to use at a reduced or no cost. A majority of websites used Facebook and Twitter as social media for sharing of online information. While many people search for information on the Internet for a family member or a friend [9], information sharing is currently powered by using social media.

Evaluating the quality of scientific information was challenging, especially with the lack of reliable and valid assessment tools. In addition, evaluating the scientific content requires a person who has extensive knowledge in the field. This paper demonstrated that the newly developed SIQ Scale has a high internal consistency and also displayed convergent validity with information credibility tools (DISCERN and HoNCode), which can be used by other researchers. Website affiliation was found to have a significant association with SIQ, usability, accessibility, trust, DISCERN, and HoNCode.

Linear regression models were used to explore the predictors for SIQ. The importance of this step is to make clinicians spend less time and effort to identify high-quality Internet resources, where no content analysis study is available. Website affiliation was able to significantly predict SIQ. Among different groups of website affiliation, governmental websites were found to be associated with the highest predicted SIQ score compared with the reference category. Credibility indicators—either DISCERN or HoNCode—were able to significantly predict SIQ after statistically controlling for website affiliation. A majority of variance in SIQ scores were explained by website affiliation and DISCERN. This finding is important because it might not only improve clinicians’ ability to identify high-quality online resources but also improve patients’ ability to find these resources by reviewing the governmental websites in light of DISCERN criteria.

Among the reviewed websites, the recommended treatment options were a reflection of the clinical uncertainty related to asymptomatic wisdom teeth [39]. Despite the lack of evidence supporting prophylactic removal of disease-free asymptomatic impacted wisdom teeth [3], there was a tendency to recommend the removal of asymptomatic wisdom teeth to prevent future problems. Conversely, some websites recommended the removal of only symptomatic third molars. Because of the uncertainty regarding asymptomatic wisdom teeth, some websites advised patients to obtain a second opinion. These findings suggested that clinicians should discuss this uncertainty with their patients before making a shared decision, because patients themselves might be confused due to conflicting information [10]. In addition, some websites were not providing patients with evidence-based information; for example, many websites recommended continuous application of ice packs postoperatively despite the best available information from randomized controlled trial evidence, which showed no significant difference on postoperative edema, pain, and trismus when compared with no intervention [40]. Clinicians have a responsibility to apply the current best evidence in the shared decision-making process to reach a decision that is ethical, and in the best interest of the patient. Although cost is known to provoke anxiety for dental patients [41], it was found to be the least reported information. This suggests that providing cost estimates on websites could be useful in avoiding/reducing potential anxiety related to treatment costs.

Among the used readability-grade estimates, the New FOG readability grade was the most powerful in predicting scientific information comprehensibility. The significant correlation between the mean readability grade and Scientific Information Comprehension Scale score suggested convergent validity and consequently that the Readability Studio software could be used to assess information comprehensibility. In this study, the estimated mean readability grade was higher than Grade 8 as recommended by some health authorities [17]. Attention should be paid to provide information in a way that is patient centered.

The strong and significant correlation between websites’ ranking according to their SIQ and their ranking according to total scoring suggested that websites associated with the SIQ were also associated with other quality aspects such as readability, usability, trust, and credibility. Such results suggest that future research might focus on the SIQ Scale, readability-grade estimate, and DISCERN to limit the evaluation process.

The main limitation of this study lay in 2 main areas: sampling bias and examiner bias that were known to the researchers when conducting data collection and analysis. However, effort was made to minimize their impact by using predetermined assessment criteria and to statistically validate the measurements used. In addition, websites were evaluated by the main author who has appropriate academic qualifications and clinical experience—an approach that has been used in previous research [42]. With regard to sampling bias, the retrieved websites were limited to the keywords that were used and search engines on a certain day.

The strengths of our study were (1) the contribution to the field of health informatics such as the development and initial validation of the SIQ Scale and the validation of Readability Studio Professional 2012; (2) contribution to current clinical practice by providing a shortlist of high-quality websites (however, clinicians need to consider the dynamic nature of online resources); (3) the development of criteria for patient information concerning wisdom tooth problems (see Multimedia Appendix 1), which might be used as an information sheet covering all areas of wisdom teeth removal and using the best available evidence; (4) use of a statistical approach to analyze website data that has not been used previously in these kind of studies, such as convergent validity, linear regression using dummy variables, and thematic analysis of open comment section using NVivo 10; and (5) the validation of a readability software application that could be used in future research. The SIQ Scale [24] demonstrated some evidence of both reliability and validity in assessing the SIQ; hence, it might be usable in future research related to the assessment of online health information.

### Conclusion

This study provides clinicians with guidance in assessing Internet resources for patients suffering from wisdom tooth problems. However, clinicians may apply similar techniques when recommending websites to patients who suffer from other dental problems. Consumer health information providers should consider evidence-based information, use of multimedia, and information readability during the process of information production. Readability Studio Professional 2012 was found to be valid as a software application for assessing comprehensibility of online health information. Website affiliation and DISCERN were found to play a major role in the prediction of SIQ. Governmental websites were associated with the highest prediction for SIQ. DISCERN and HoNCode as online information credibility tools were significantly able to predict the SIQ. In instances where no guidance is available, patients could review governmental websites in light of DISCERN criteria to identify high-quality information. The developed SIQ Scale had high internal consistency and established convergent validity, suggesting its use in the future to assess the SIQ of online dental information.

## Appendix

Multimedia Appendix 1 Criteria used for evaluating scientific information quality concerning wisdom teeth problems.

## Abbreviations

ANOVA: analysis of variance
HoN: Health on the Net
HoNCode: Health on the Net Code
SIQ: scientific information quality
SI Comprehension: Scientific Information Comprehension
SI Referencing: Scientific Information Referencing
SI Reporting: Scientific Information Reporting
TMs: third molars
