# Supplementary material for: Third Molars on the Internet: A Guide for Assessing Information Quality and Readability
Source: Interact J Med Res. 2015 Oct 6;4(4):e19. doi: 10.2196/ijmr.4712 (PMC4704926; doi:10.2196/ijmr.4712)
Supplement: Multimedia Appendix 1 [file ijmr_v4i4e19_app1.pdf]

Appendix 1

Criteria used for evaluating scientific information quality concerning wisdom teeth problems

Kamal Hanna[1], David Brennan[2], Paul Sambrook[3], Jason Armfield[4]

[1]Kamal Hanna, BDS, MSc (oral Surg.), PGDip (laser), MRCSEd (oral Surg.), Egypt Board OMFS.

PhD Candidate, Australian Research Centre for Population Oral Health (ARCPOH), University of Adelaide

[2]David Brennan, BA (Hons), Grad. Dip (CIS), MPH, PhD.

Professor, Australian Research Centre for Population Oral Health (ARCPOH), University of Adelaide, Adelaide, South Australia, Australia.

[3]Paul Sambrook, M.B.B.S., M.D.S., F.R.A.C.D.S. OMS

Consultant OMFS, Adelaide Dental Hospital, University of Adelaide, Adelaide, South Australia, Australia.

[4]Jason Armfield, BA (Hons), PhD.

Associate Professor, Australian Research Centre for Population Oral Health (ARCPOH), University of Adelaide, Adelaide, South Australia, Australia.

Corresponding Author

Kamal Hanna

Australian Research Centre for Population Oral Health

122 Frome St., Adelaide, 5005, South Australia, Australia

Phone: +61(08) 8313 5626

Email: kamal.hanna@adelaide.edu.au

Overview

The wisdom tooth (third molar) is the last tooth to appear in the mouth usually in the age range between 17-25 years. Each individual normally has four wisdom teeth, one at the back of each quadrant of your mouth. Due to its late eruption, often there is a lack of enough room to accommodate them. When they are prevented from reaching their functional position in the mouth, they become “impacted”. The majority of young adults have one or more impacted wisdom teeth [1].

Presentation

During the period when they erupt into the mouth, few patients (about 10%) may develop some minor discomfort while “teething”. If they reached their functional position in your mouth and you maintain your oral hygiene, they are like your back teeth (molars). In a situation where your wisdom tooth attains an abnormal position or there is some difficulty in cleaning them, you may get some gum diseases or decay which may result in pain or recurrent infections, swelling and limitation of your mouth opening. If you have a treatment to straighten your teeth, your orthodontist may decide that, your wisdom teeth might interfere with your orthodontic treatment. However, the best available evidence does not support the responsibility of impacted wisdom teeth for late teen crowding [2,3]. Impacted wisdom teeth are associated with other conditions (12%) such as resorption of roots of adjacent teeth, fluid filled sac (Cyst) or tumors [4].

Diagnosis

Your dentist will be able to advise you about your wisdom teeth or s/he might refer you to an oral surgeon. Your dentist/oral surgeon will need to get your medical and dental history, examine you and request some X-rays (images) of your wisdom teeth – normally a panoramic dental image is

sufficient. In some situations, more sophisticated images might be requested depending on the position of your wisdom teeth, and how close they are to important structures [5].

Treatment Options

The decision about a treatment choice is made after thorough evaluation, providing you with information for the available treatment options, risks/ benefits, giving you the opportunity to discuss such information and considering your opinion, before finally reaching a shared decision. In general, if your wisdom teeth are erupted in their normal position, and they do not cause any problem, then there is no need for them to be removed. Symptomatic (problematic) wisdom teeth, either impacted or not, that are associated with pathology or interfere with orthodontic treatment should be removed. If your wisdom teeth are impacted but disease free, the current best available evidence does not support their removal to prevent future problems [2,3,6] If a decision was made to retain the impacted wisdom tooth/teeth, your oral surgeon may decide to actively monitor them. Complete surgical removal is the commonly used treatment. In some situations where there is high risk if the impacted tooth is completely removed, coronectomy (removing the crown and retaining the roots) might be considered [7].The majority of surgical removals of third molars are carried out under local anesthetic in clinics and can be carried out either by your dentist or specialist oral surgeon. However, if you have a history of certain medical problems, your surgeon might decide to refer you to a hospital to have it done there. For patients who have needle phobia or dental anxiety, please refer to anxiety control information section.

Risk and Benefits

Surgical removal of impacted wisdom teeth is associated with some risks. Such risks include pain, bleeding, swelling, limitation of mouth opening and infection. These general risks are temporary and will disappear by the first post-operative week [8], and can be controlled by medications and/or

instructions that will be provided to you by your oral surgeon. Surgical removal of lower wisdom teeth is associated with a risk of developing numbness (tingling) of the lower lip and/or the side of your tongue. These risks are associated with how close your lower wisdom tooth is to nerves that supply them with sensation. This tingling sensation occurs due to pressure or injury to these nerves during surgery. Only 0.4-8.4% of people who have the surgery develop this sensation temporarily and this only may last for few days to several weeks [3]. However, in very few cases (up to 1%), this sensation may last longer and become permanent. For the upper wisdom tooth, surgical removal is associated with a risk of developing a communication between your mouth and your nasal sinus. If this happens, the majority will heal by itself with some precautions such as the use of nasal inhalation and avoid nose blowing. However, in a few cases, surgical closure might be considered.

The benefit of removing the problematic wisdom tooth is preventing future problems. However, there is a lack of evidence in the current research that supports the prophylactic removal of non-problematic disease free impacted wisdom teeth [1,2,3]. The fate of wisdom tooth cannot be predicted, and the incidence of disease free wisdom teeth to develop a cyst/ tumor is as low as 1-2% [3].

The procedure of surgical removal of impacted wisdom tooth

After a decision is made for removing your wisdom teeth, your dentist/oral surgeon will need to get your consent for the procedure. This consent is to ensure that you know which teeth are going to be removed, risks/ benefits and post-surgery complications that might occur. An appointment will be given to you for this procedure. You will be given a local unaesthetic injection which is the same as you have when you receive a filling to numb the area up so you do not feel any pain. After your dentist/ oral surgeon makes sure that the area of surgery is completely numbed, your dentist can start the procedure of surgical extraction. Depending on the position of your wisdom tooth, your wisdom tooth extraction can be like any other dental extraction.

If your wisdom tooth is impacted, your dentist/oral surgeon will need to make a cut in your gum to get access to the tooth. The procedure may require your dentist/oral surgeon to use the drill to cut bone around the tooth and/or section the tooth into smaller pieces to facilitate its removal. During this process, you may feel some pressure or vibration. In each case, no pain should be felt. If you feel any unpleasant sensation, raise your hand to your oral surgeon/dentist so they can give you more anesthetic. After the tooth is removed, the area will be flushed and inspected for any sharp edges or remaining tissues. Your dentist/oral surgeon might stitch your gum usually with dissolving stitches. You will be asked to gently bite on a moist gauze pad for few minutes till the bleeding stops [9].

Post-Operative Care and Recovery

After your surgery, you will be given time to rest in the recovery room to make sure that the bleeding has stopped and you are able to stand and walk. During this time, post-surgery instruction, emergency contacts and/or medication will be provided to you before letting you go home. Also, you might be offered a follow-up appointment. It is preferable if you have escort to drive you home on the day of surgery. If you have your surgery done under general anesthesia (GA) or sedation, you are not allowed to drive.

How Can You Control Post-Operative Complications?

Pain

You will need to take the prescribed analgesics before your anesthetic wears off. You should take your prescribed medication, as instructed, even if you do not feel any problems. Some surgeons use long acting local anesthesia to avoid the peak of post-operative pain, which usually occurs four hours after the surgery. Usually a combination of paracetamol and ibuprofen is effective in relieving pain [10]. However, you should not exceed the daily recommended dose. Your dentist/oral surgeon

may prescribe narcotic analgesics. Pain usually decreases with time, and will disappear five days after your surgery. If your pain increased after the first 48 hours following your surgery, it could be a sign of localized inflammation.

Bleeding

After the surgery, you will be expected to have some oozing from the extraction socket. Therefore, you may find your pillow is stained with blood. You should not exercise, drink hot drinks, eat hot food or drink using a straw in the first 24 hours. Try to raise your bed up by 45 degrees to decrease oozing of blood. However, if you notice fresh blood, you can gently bite on a moist gauze pad for a few minutes till active bleeding stops. You can also consider biting gently on a moist tea bag. If these instructions do not stop the bleeding, then you need to contact your dentist/ oral surgeon as per the post-operative instruction sheet provided.

Swelling

Post-operative swelling depends on many factors, such as surgical difficulty, surgical technique, gender and fat distribution. The swelling reaches its maximum after 48 hours, and gradually decreases with time to reach normal by the 5th to7^{th} day after your operation. Despite some surgeons recommending the continuous application of ice-packs, evidence shows no difference in swelling [11] if ice-packs were not used.

Dry/ Infected Socket

Dry and infected socket are localized complications that might occur after surgical extraction. Dry socket occurs when the formation of blood clot in the socket is prevented by vigorous mouth rinsing, suction of fluids through a straw or smoking. This leads to exposure of nerve endings in jaw bones, which is extremely painful. In the case of an infected socket, the blood clot lyses due to microbial products; and could be treated by irrigating the extraction socket to clean debris and insert

a dressing [12]. This management needs to be repeated by your dentist till your symptoms disappear. In some situations, antibiotics might be prescribed.

Infection

Infection after your surgery may occur and it is manifested by increased pain, swelling, bad taste and odour [13]. If you think that you developed infection, you should contact your oral surgeon. Your oral surgeon may prescribe you antibiotics. Some surgeons prefer to prescribe antibiotics, as a prophylactic, after the surgery to prevent post-operative infection. However, the best available evidence questions the use of prophylactic antibiotics, as they do more harm than good [14].

Your Diet

In the first 24 hours after your surgery, you are advised to have a cold soft diet such as ice-cream, yogurt, jelly or pudding. This diet is gentle on your gums and reduces blood oozing from the extraction site. Try to avoid eating on the operation site for the first few days. After the first post-operative day, you can start having semi-solid diet such as mashed potatoes, eggs or pasta. Avoid rice as it can be trapped under your gums and cause discomfort. You will gradually return to your normal diet after the first post-operative week. Alcoholic beverages should be avoided for at least 24 hours especially if you had general anesthesia/ IV sedation, until you stop taking your prescribed analgesics and antibiotics. Smoking should be avoided for the first post-operative week, as it might lead to dislodgement of the formed blood clot and/or delay wound healing.

Anxiety Control and Sleep Dentistry

In some cases you may feel dental anxiety, fear or phobia; you need to discuss these issues with your dentist/oral surgeon. Your dentist will carry out an assessment process, in order to advise you with the best anxiety control option. There are different techniques that might be used for this purpose. These techniques are ranging from behavioral change, hypnosis to the use of medications.

In the case of medications, you need to follow the instructions of your dentist. The levels of anxiety control using medications are:

Conscious sedation

During conscious sedation, you will feel drowsy, but you will be able to communicate with your dentist/oral surgeon, breath normally and maintain your reflexes. There are three types of conscious sedation techniques that are commonly used. This technique provides different sedation depth depending on drug type, dose and route of delivery. These techniques are [15]:

Oral conscious sedation

This sedation is carried out using medication taken by mouth (the same as sleeping pills) which will be provided to you by your dentist/oral surgeon to be taken the night before the surgery and/or shortly before the surgery.

Gas sedation

This type of conscious sedation uses gas inhalation, usually laughing gas that is delivered to you through a mask on your nose.

Intra-venous sedation

A sedative medication will be delivered to you via a small catheter in your hand vein. If you have a needle phobia, ask your dentist/oral surgeon to numb the skin of your hand before the insertion of the intravenous catheter.

General anesthetic (GA)

During general anesthesia, you will be completely asleep. GA will be delivered to you by an anaesthetist. GA needs to be performed at a hospital, normally as a day case. In some situations,

patients may be admitted to hospitals, depending on the complexity of their medical history and the procedure [16].

Cost

Wisdom tooth surgery is associated with direct and indirect cost. Direct cost includes the dentist/ surgeon fee. This cost increases if it is associated with sedation or GA, when anesthetist and hospital/theatre fees are included. The cost you will pay depends on the health system and your insurance status. Check with your dentist/ oral surgeon for more information. The indirect cost includes time off work after the surgery, normally 1.5 – 3 days depending on the surgical difficulty and your ability to recover.

References

1. Dodson T, Susarla S. Impacted wisdom teeth. *Clin Evid (Online).* 2010;2010(4):1302. PMID: 21729337

2. Mettes T, Ghaeminia H, Nienhuijs M, Perry J, van der Sanden WJ, Plasschaert A. Surgical removal versus retention for the management of asymptomatic impacted wisdom teeth. *Cochrane Database Syst Rev.* 2012;6:CD003879. PMID: 22696337

3. Kandasamy S, Rinchuse D, Rinchuse D. The wisdom behind third molar extractions. *Aust Dent J.* Dec 2009;54(4):284-292. PMID: 20415925

4. Friedman J. The prophylactic extraction of third molars: a public health hazard. *Am J Public Health.* Sep 2007;97(9):1554-1559. PMID: 17666691

5. Roeder F, Wachtlin D, Schulze R. Necessity of 3D visualization for the removal of lower wisdom teeth: required sample size to prove non-inferiority of panoramic radiography compared to CBCT. *Clin Oral Investig.* Jun 2012;16(3):699-706. PMID: 21519882

6. Dodson T, Susarla S. Impacted wisdom teeth. *Clin Evid.* 2010;2010PMID: 21729337

7. Geisler S. Coronectomy is an effective strategy for treating impacted third molars in close proximity to the inferior alveolar nerve. *J Am Dent Assoc.* October 1, 2013 2013;144(10):1172-1173. PMID: 24080934

8. Sancho-Puchades M, Valmaseda-Castellon E, Berini-Aytes L, Gay-Escoda C. Quality of *Bucal.* Nov 2012;17(6):e994-999. PMID: 22926461

9. Ness G, Peterson L. Peterson's principles of oral and maxillofacial surgery. In: Miloro M, ed. *Peterson's principles of oral and maxillofacial surgery.* Vol 1. 2nd ed. Canada: BC Decker Inc; 2004.

10. Mehlisch D, Aspley S, Daniels S, Southerden K, Christensen K. A single-tablet fixed-dose combination of racemic ibuprofen/paracetamol in the management of moderate to severe postoperative dental pain in adult and adolescent patients: a multicenter, two-stage, randomized, double-blind, parallel-group, placebo-controlled, factorial study. *Clin Ther.* Jun 2010;32(6):1033-1049. PMID: 20637958.

11. van der Westhuijzen A, Becker P, Morkel J, Roelse J. A randomized observer blind comparison of bilateral facial ice pack therapy with no ice therapy following third molar surgery. *Int J Oral Maxillofac Implants.* May 2005;34(3):281-286. PMID: 15741037

12. Chemaly D. How do I manage a patient with dry socket? *J Can Dent Assoc.* 2013;79:d54. PMID: 23763736

13. Blondeau F, Daniel N. Extraction of impacted mandibular third molars: postoperative complications and their risk factors. *J Can Dent Assoc.* May 2007;73(4):325. PMID: 17484797

14. Lodi G, Figini L, Sardella A, Carrassi A, Del Fabbro M, Furness S. Antibiotics to prevent complications following tooth extractions. *Cochrane Database Syst Rev.* . 2012;11:Cd003811. PMID: 23152221

15. O'Halloran M. The use of anaesthetic agents to provide anxiolysis and sedation in dentistry and oral surgery. *Australas Med J.* 2013;6(12):713-718. PMID: 24391684

16. Academy of Medical Royal Colleges. 2013. *Safe sedation practice for healthcare procedures* [Online]. Available: http://www.aomrc.org.uk/doc_view/9737-safe-sedation-practice-for-healthcare-procedures-standards-and-guidance Archived at: http://www.webcitation.org/6RfW8AQKZ.
